# Work-family behavior role conflict affect the health outcomes of emergency department nurses: gender difference study by propensity score matching

**DOI:** 10.3389/fpubh.2025.1679370

**Published:** 2026-01-14

**Authors:** Luying Zhong, Jing Zhou, Hao Zhang, Ling Zhu, Dongmei Diao, Jianna Zhang, Xiaoli Chen

**Affiliations:** ^1^Department of Emergency Medicine, West China Hospital, Sichuan University/West China School of Nursing, Sichuan University, Chengdu, China; ^2^Disaster Medical Center, Sichuan University, Chengdu, China; ^3^Nursing Key Laboratory of Sichuan Province, Sichuan University, Chengdu, China

**Keywords:** emergency department nurse, gender differences, physical symptoms, propensity score matching, psychological symptoms, sleep disorders, work–family behavior role conflict

## Abstract

**Background:**

Emergency department nurses work in environments with high stress and irregular shifts, which poses a high risk of work–family behavior role conflict (WFBRC). Conflicts have a negative impact on health, including psychological symptoms like anxiety and depression, along with physical symptoms like fatigue, and sleep disorders. The scores of different dimensions of WFBRC vary among different genders, and it is unknown whether there are differences in terms of the impact of different dimensions of WFBRC on health problems among nurses of different genders.

**Aim:**

To investigate the impact of WFBRC on the emergency department nurses’ health outcomes by comparing different gender.

**Study design:**

Secondary analysis of a cross-sectional study. A stratified cluster group sampling method was employed to survey emergency department nurses from 30 hospitals in China. Data from the Work-Family Behavioral Role Conflict Scale (WFBRC-S), the Self-assessment scale of somatization symptoms (SSS-CN), and a self-administered sleep questionnaire (SSQ) were collected via electronic questionnaire format, and propensity score matching (PSM) was employed to balance sociodemographic factors, and then a linear regression analysis was performed to clarify gender differences.

**Results:**

A total of 1,540 emergency department nurses were surveyed, and dataset of 329 male and female emergency department nurse pairs were successfully generated. The study showed male emergency department nurses report higher family-to-work conflict (FWC) scores (25.93 ± 11.85) than females (23.36 ± 10.55). Work-to-family conflict (WFC) has a greater impact on the physical and psychological symptoms of female emergency department nurses (*β* = 0.607) than male nurses (*β* = 0.392), while FWC shows the opposite pattern [female (*β* = 0.165) VS male (*β* = 0.351)]. Regarding sleep disorders, WFC affects female nurses (*β* = 0.460) is close to that of male nurses (*β* = 0.467).

**Conclusion:**

Male emergency department nurses report higher FWC scores than females. WFC has a greater impact on the physical and psychological symptoms of female emergency department nurses than male nurses, while FWC shows the opposite pattern.

## 1 Introduction

In recent years, the issue of work–family behavior role conflict (WFBRC) had become increasingly important in the fields of occupational health and nursing research. WFBRC refer to a state of imbalance between an individual’s work and family roles. WFBRC have a nonnegligible correlation with nurses’ quality of life (1), well-being (2), and physical and psychological health (3), and it is a significant influencing factor in nurse turnover (4, 5). These conflicts have been shown to have a profound effect on employee well-being and health outcomes, which is exacerbated by the nature of emergency department nurses’ work (6). Emergency department nurses play a vital role in the healthcare system and are often faced with heavy workloads and high pressure, demanding work environments that require more labor as well as rapid response and decision-making in the face of life-threatening situations. Long and irregular working hours, shift work, sudden increases in patients, and the emotional toll of dealing with critical cases (7–9) encroach on their time and energy for family matters. At the same time, they are also indispensable members of the family whose responsibilities and roles are not only in the workplace but also in nursing children and taking care of their families, which will also affect their ability to concentrate and perform at their best at work and put emergency department nurses through a double ordeal (10).

High family-to-work conflict (FWC) and work-to-family conflict (WFC) are associated with poorer physical performance and mental health (11–14), inducing mental health problems such as anxiety depression and causing psychological distress in both men and women (15, 16) and moderating the occurrence of work-related skeletal muscle pain and stress (17). Individual responses to conflict generation in daily life have also been associated with poorer sleep quality and higher levels of psychological distress (18), with higher levels of WFBRC tending to imply poorer sleep quality. In addition, as a prevalent form of depression among healthcare workers (19), WFC and FWC were also significantly negatively correlated with depression symptoms, and it is worth noting that sleep disturbance had a 40.54% mediating role in translating WFBRC into depressive symptoms (20, 21), while sleep quality and depressive status explained 20.2% of nurses’ WFBRC scores (22), with depression and sleep-related problems mutually and jointly influencing WFBRC. This suggests that there is an interaction effect between WFBRC and health outcomes. Addressing WFBRC are a legitimate means of promoting the physical and mental health of nurses in the emergency department, and addressing all the risks arising from work-life interactions is important.

However, gender has a significant impact on nursing based on historical and societal factors (23). Historically, gender roles in society have placed different expectations on caring for family and work responsibilities. Female nurses may face additional pressures associated with traditional gender norms, such as taking on more household and parenting responsibilities (24). In a female-dominated profession, due to the influence of social stereotypes (25), male nurses may encounter unique challenges in balancing their career aspirations with family expectations. Despite the growing body of research on WFBRC and its impact on nurses, relatively little research has addressed gender-specific performance situations and perceived conflict-generated health outcomes response to WFBRC among emergency department nurses. Understanding these dynamics is critical, as it can inform targeted interventions and support strategies to improve the health and job satisfaction of emergency department nurses, ultimately contributing to improved patient care and a more sustainable healthcare workforce.

According to the Work Family Conflict Theory model, conflicts arise from the individual’s role conflict, boundary control, and differences in gender role expectations (26). Family responsibilities and organizational management can exacerbate the level of conflict, affecting the health of the individual and the family, and ultimately affecting job performance. There are partial differences in the roles assumed by different genders in the home and work environments, and it is not yet known whether there are differences in the impact of different dimensions of WFBRC on the health status of emergency department nurses of different genders. Therefore, this study aims to comprehensively explore the effects of different dimensions of WFBRC on the health status of emergency department nurses of different genders, to provide valuable references and insights for healthcare administrators, policy makers, and nursing professionals, and to assist administrators in making more informed decisions and formulating targeted strategies to better safeguard the health of emergency department nurses and optimize the entire healthcare work environment.

## 2 Method

### 2.1 Study population

A stratified cluster sampling method was employed: 3 to 5 hospitals were randomly selected from each of China’s seven major geographic regions (North China, Northeast China, East China, Central China, South China, Southwest China, and Northwest China). The final survey covered emergency department nurses from 30 hospitals during the period from December 26, 2023, to January 18, 2024. In each participating hospital, all registered nurses were invited to take part in the study. The inclusion criteria were as follows: ① licensed nurses in the emergency department and ② had ≥ 1 year of emergency work experience. The exclusion criteria were as follows: ① had a history of psychiatric disorders; ② were nurses on advanced training; and ③ were nurses on sick leave and breastfeeding (≥1 month). It is important to note that participation was voluntary, and all eligible nurses provided informed consent before their inclusion in the study. This study has been approved by the Ethics Review Committee of West China Hospital, Sichuan University [Approval No. 2024(309)].

### 2.2 Research methodology

#### 2.2.1 Research tools

##### 2.2.1.1 General information questionnaire

The research team itself includes 10 variables, namely, age, gender, marital status, reproductive status, highest education, job title, years of working experience, weekly working hours, night shift status and monthly income.

##### 2.2.1.2 Work-Family Behavioral Role Conflict Scale (WFBRC-S)

The source scale used in this study was the Work-Family Behavioral Role Conflict Scale (WFBRC-S) developed by Clark et al. (27), and the Chinese version of the Work Family Behavioral Role Conflict Scale (WFBRC-S) revised by Sun et al. (28). The scale divides work–family behavioral role conflict into two dimensions, work family behavioral role conflict (WFC) and family work behavioral role conflict (FWC), with a total of 19 items. The participants’ response to each item is the frequency of occurrence of each behavior, which ranges from 1 (never) to 5 (very frequently), and the higher the score is, the higher the frequency of WFBRC among emergency department nurse. The scale has a Cronbach’s *α* coefficient of 0.969 with good reliability.

##### 2.2.1.3 Self-assessment scale of somatization symptoms (SSS-CN)

The Chinese version of the Somatization Symptoms Self-Rating Scale (SSS-CN) developed by Jiang et al. (29) was used, which consists of 20 short items consisting of four dimensions: physical symptoms, anxiety, depression, and anxiety-depression. Participants responded to each item as to how well each behavior was present or tolerated, ranging from 1 (nonexistent) to 4 (present almost every day or harder to tolerate). Higher scores indicate more severe symptoms, and in this study, anxiety and depression were combined and analyzed as psychological symptoms. The Cronbach’s alpha coefficient for this scale in this study was 0.974, indicating good reliability.

##### 2.2.1.4 Self-administered sleep questionnaire (SSQ)

The SSQ (30) is used to assess sleep disorders in emergency department nurses, and three categories of sleep symptoms, namely, time to fall asleep, persistent sleep, and early morning awakening, are evaluated by three items; the higher the score is, the more serious the degree of sleep disorders. The Cronbach’s alpha coefficient of this scale was 0.809, indicating good reliability.

#### 2.2.2 Data collection and organization

The Questionnaire Star was used to develop an electronic questionnaire with an informed consent form. All questionnaire items were made mandatory to ensure completeness, and each IP was limited to responding only once to prevent duplicate responses. Prior to the survey, a core person in charge of each hospital was designated and trained in the key concepts and guidelines of the survey so that they could explain the survey content accurately and consistently. When the survey was conducted, an electronic link was pushed to the respondents through the WeChat software, the questionnaire began with an explanation of the purpose of the study, and the respondents were surveyed on their willingness to fill it out, and only after the respondents had read the informed consent form and agreed to participate could they enter the question-and-answer interface, and if they had any questions, they could consult the core person in charge of the hospital to which they belonged.

#### 2.2.3 Statistical analysis

All data were statistically analyzed by SPSS 26.0 statistical software. First, categorical variables were analyzed using component ratios (%) and continuous variables were analyzed using descriptive statistics using arithmetic means (M) and standard deviations (SD). Subsequently, propensity score matching (PSM) was used to obtain two datasets with balanced distributions of confounders. In this study, age stratification, marital status, fertility status, education attainment, professional title, years of working experience, weekly working hours, frequency of night shifts and monthly income were used as covariates, and the PSM method was used to match the covariates 1:1, setting the caliper value of 0.02, and finally resulting in the matched dataset. To compare whether there was any difference between the two datasets of men and women in terms of the impact of WFBRC on the health outcomes of nurses in the emergency department after the confounders were balanced, linear regression analysis was used, with a test level of *α* = 0.05. Confirm the normality of data distribution using residual histograms, and assess multicollinearity using tolerance and variance inflation factor (VIF) values.

## 3 Results

### 3.1 PSM results

A total of 1,540 emergency department nurses, 1,211 females and 329 males, were surveyed in this study. Finally, 329 data pairs were matched. A comparison of the general information of the datasets of the matched male and female groups is shown in Table 1, and none of the differences were statistically significant (*p* > 0.05), with better control of confounders in both groups. Table 2 describes the results of the variables related to the study after PSM and shows that the differences between male and female nurses were not statistically significant except for FWC, where male nurses had higher FWC cores than female nurses.

**Table 1:** Demographic characteristics after PSM.

Variables		Male	Female	*χ* ^2^	*p*
Age stratification	20–29 years old	177	172	2.418	0.510
	30–39 years old	147	149		
	40–49 years old	4	8		
	≥50 years old	1	0		
Marital status	Unmarried	166	155	0.736	0.435
	Married	163	174		
Fertility status	Infertile	205	192	1.073	0.339
	Fertile	124	137		
Education attainment	Junior college	51	51	0.000	1.000
	Undergraduate and above	278	278		
Professional title	Junior	234	229	0.388	0.816
	Middle	91	97		
	High	4	3		
Stratification by years of working experience	≤5 year	157	148	2.164	0.535
	6–10 year	111	126		
	11–15 year	56	48		
	16–20 year	5	7		
Weekly working hours	<40 h	112	107	2.675	0.446
	41–48 h	169	184		
	49–58 h	31	21		
	≥59 h	17	17		
Frequency of night shifts	0 times/month	16	16	0.000	1.000
	1–4 times/month	58	58		
	5–8 times/month	144	144		
	≥9 times/month	111	111		
Monthly income	<4000RMB/month	16	10	4.146	0.390
	4,000-5999RMB month	31	32		
	6,000-7999RMB/month	47	56		
	8,000-9999RMB/month	81	94		
	≥10000RMB/month	154	137		

**Table 2:** Comparison of study-related variables after PSM.

Variables	Male	Female	*t*	*p*
WFC scores	17.48 ± 7.22	17.75 ± 6.98	−0.940	0.621
FWC scores	25.93 ± 11.85	23.36 ± 10.55	2.932	0.003
Physical symptoms scores	18.52 ± 7.08	18.85 ± 6.97	−0.616	0.538
Psychological symptoms scores	19.68 ± 7.35	20.19 ± 7.22	−0.906	0.365
Total symptom scores	38.2 ± 14.15	39.05 ± 13.81	−0.778	0.437
Sleep disorders scores	8.32 ± 3.22	8.27 ± 3.10	0.185	0.853

### 3.2 Results of the analysis of the correlation between family work conflicts and health outcomes

The results of this study showed that there was a correlation between WFC and FWC with physical symptoms, psychological symptoms, and sleep disorders, with correlation coefficients ranging from 0.354 to 0.719. Overall, WFC and FWC correlated well with physical symptoms, psychological symptoms, and generally correlated with sleep disorders, and all differences were statistically significant. See Table 3 for details.

**Table 3:** Analysis of the correlation between WFBRC and health outcomes.

Variables		Health outcomes			
		Physical symptoms	Psychological symptoms	Somatization symptoms score	Sleep disorders
Total	WFC	0.647**	0.718**	0.697**	0.518**
	FWC	0.606**	0.603**	0.617**	0.417**
Female nurse	WFC	0.662**	0.747**	0.719**	0.488**
	FWC	0.559**	0.573**	0.578**	0.354**
Male nurse	WFC	0.631**	0.691**	0.675**	0.546**
	FWC	0.662**	0.646**	0.667**	0.474**

**Significant correlation at the 0.01 level (two-tailed).

### 3.3 Results of the analysis of the impact of WFBRC on health outcomes

After controlling for confounding factors through PSM, a linear regression analysis was performed with health outcomes as the dependent variable and WFC and FWC as independent variables. The residual histograms from all linear regression analyses approximate a normal distribution. Furthermore, all tolerance values exceeded 0.1 and VIF values were below 10, indicating no evidence of multicollinearity.

#### 3.3.1 Total emergency department nurses

For every 1 increase in the WFC score, the total somatization symptoms score increased by 1.044, of which the physical symptoms score increased by 0.432, the psychological symptom score increased by 0.612, and the sleep disorders score increased by 0.206. For every 1 increase in the FWC score, the total somatization symptoms score increased by 0.279, of which the physical symptoms score increased by 0.176, and the psychological symptom score increased by 0.102. The effect of FWC on sleep disorders score was not statistically significant. See Table 4 for details.

**Table 4:** Impact of WFBRC on health outcomes in emergency department nurses.

Implicit variable	Independent variable	Total		Female		Male	
		*B*	Beta	*B*	Beta	*B*	Beta
Physical symptoms score	(Constant)	6.726		6.433		6.959	
	WFC	0.432	0.437	0.523	0.525	0.274	0.280
	FWC	0.176	0.283	0.134	0.203	0.261	0.437
Psychological symptoms score	(Constant)	6.644		6.266		6.936	
	WFC	0.612	0.600	0.677	0.665	0.495	0.486
	FWC	0.102	0.160	0.082	0.121	0.158	0.255
Somatization symptoms score	(Constant)	13.370		12.699		13.895	
	WFC	1.044	0.530	1.201	0.607	0.769	0.392
	FWC	0.279	0.225	0.216	0.165	0.419	0.351
Sleep disorders score	(Constant)	4.149		4.354		3.983	
	WFC	0.206	0.463	0.205	0.460	0.208	0.467
	FWC	0.021	0.074^*^	0.012	0.042^*^	0.027	0.099^*^

^*^p > 0.05.

#### 3.3.2 Female emergency department nurses

For every 1 increase in the WFC score, the total somatization symptoms score increased by 1.201, of which the physical symptoms score increased by 0.523, the psychological symptoms score increased by 0.677, and the sleep disorders score increased by 0.205. For every 1 increase in the FWC score, the total somatization symptoms score increased by 0.216, of which the physical symptoms score increased by 0.134 and the psychological symptoms score increased by 0.082. The effect of FWC on sleep disorders scores was not statistically significant. See Table 4 for details.

#### 3.3.3 Male emergency department nurses

For every 1 increase in the WFC score, the total somatization symptoms score increased by 0.769, of which the physical symptoms score increased by 0.274, the psychological symptoms score increased by 0.495, and the sleep disorders score increased by 0.208. For every 1 increase in the FWC score, the total somatization symptoms score increased by 0.419, of which the physical symptoms score increased by 0.261, and the psychological symptoms score increased by 0.158. The effect of FWC on sleep disorders scores was not statistically significant. See Table 4 for details.

## 4 Discussion

The results of this study showed that after controlling for confounding factors such as age stratification, the difference between male and female emergency department nurses’ WFC was not statistically significant, whereas male nurses had greater FWC than female nurses did, which seems to be an interesting reversal of the traditional Chinese concept. After matched one-to-one for age, title, years of service, and so on, male nurses had greater FWC, unlike Al-Hammour’s findings. Gender moderates the relationship between risk propensity and FWC, with risk propensity significantly influencing FWC among male but not female nurses, and FWC increases as male nurses’ risk propensity increases (31). Although the limited number of relevant studies on risk propensity restricts our further discussion, in the context of China’s national conditions and male personality traits, men are more daring to take risks at work, and family ties are more likely to affect work, which in turn produces greater WFBRC than female nurses do, as well as women’s greater level of emotional support in the family, resulting in lower WFBRC (32). The results of another cross-temporal meta-analysis revealed that, in recent years, male and female nurses in China have been more likely to take risks at work than female nurses (33). The analysis revealed that both male and female employees in China have shown an increasing trend in WFBRC in recent years, with a slightly more pronounced increase among male employees, and all of the above findings are part of the potential possibilities for high WFBRC scores among male nurses. The overall scores are also different from the previous common perception that nurses experience more WFC than FWC do (34–36), but FWC and WFC are essentially two-way influences. The results of the study may be affected by nurses’ family and work situations at the time, and the time of this study was centered around a major traditional Chinese festival—Chinese New Year, which aggravated the nurses’ desire for family reunion, which may be the potential reason for the high FWC scores.

Consistent with previous findings (37–39), WFBRC was associated with health outcomes regardless of gender. It is a normal physiological response for humans to develop physical and psychological symptoms and sleep disturbances in the face of the stimulus of WFBRC. The results of this study indicate significant gender differences in the impact of WFC and FWC on the physical and mental health of emergency department nurses. Specifically, WFC has a greater impact on the physical and mental well-being of female nurses, while FWC affects male nurses more significantly. This is not only related to the deep-rooted influence of Chinese socio-cultural norms but is also affected by the high-pressure nature of emergency nursing work. In recent years, rising female labor force participation in China (40) and greater awareness of women’s career development have led more women to enter diverse professional fields. Nevertheless, traditional socio-cultural attitudes in China continue to impose greater childcare and household responsibilities on women, even amid progress toward gender equality. As women advance in their careers, they must devote more energy to their work (41), which typically leads to higher levels of WFC (42). From a physiological adaptation standpoint, female nurses may also exhibit relatively lower tolerance for the high intensity and shift work common in emergency departments (43), rendering them more vulnerable to the adverse effects of WFC (44). On the other hand, higher societal expectations for men’s career achievements in China, combined with potential professional biases against male nurses within the field (45), compel male nurses to dedicate greater time and energy to their work. Within the traditional Chinese family structure, men often assume fewer daily household responsibilities, as these duties are typically managed by female family members. Consequently, they lack established buffer mechanisms and coping experience when encountering FWC, which renders its impact on their health particularly pronounced.

The results of this study show that WFC is an independent influence on sleep disorders among male and female nurses in the emergency department and that the contributions of WFC is close to each other. WFBRC are reflected in the unconscious infiltration of work content into family life, which interferes with nurses’ sleep cycle and affects sleep quality, which in turn prevents them from effectively recovering from physical and mental exhaustion. A significant positive correlation was found between WFC and sleep disorders in worker and nurse populations (46), and WFC related to time and fatigue was also associated with sleep disorders (47). WFC was positively correlated with burnout in the nurse population, and sleep disorders even fully mediated the association between WFC and burnout (46). Some studies have also suggested a weak and nonsignificant association between WFBRC and sleep disorders (48), which may be related to the different demographic characteristics of the investigated population. Sleep disorders are not only the end point but also the “midpoint” of health problems arising from WFBRC, which is an important mediator between work family conflicts and physical and mental health problems in the nurse population. This association is not unique to the nursing profession but is common to all walks of life (21, 49), which further confirms the harmful effects of WFBRC on nurses. The results of this study show that WFC affects sleep disorders to a similar extent in male and female nurses, which may be related to the homogenization and de-gendering of nursing in the emergency department, where there is no difference in the content of the work faced by male and female nurses. In addition, sleep disorders among emergency department nurses are highly correlated with shift frequency, and this study has controlled for night shift frequency (50), a confounding factor, as a potential reason for the absence of differences. The detrimental effects of WFBRC on nurses emphasize the importance of nurses’ self-adjustment and nursing managers’ assistance in maintaining work family balance.

## 5 Limitations and suggestion

This study has several limitations. First, the cross-sectional design precludes establishing causal relationships between WFBRC and health outcomes in emergency department nurses. Although associations were observed, the temporal sequence remains uncertain. Second, as all data originated from Chinese hospitals, the findings may have limited generalizability to other cultural or healthcare settings. Finally, the reliance on self-reported measures introduces the potential for response biases, including social desirability and recall bias. Future research could use longitudinal designs to explore causal links between WFBRC and emergency nurses’ health outcomes, expand samples across diverse settings to enhance generalizability, and supplement self-reported data with objective measurements to mitigate response biases.

## 6 Recommendations or implications for practice

This finding underscores the importance of gender-tailored interventions in clinical settings. Given that male emergency department nurses experience higher FWC, healthcare managers should consider flexible scheduling and family support programs to alleviate this stressor. For female nurses, addressing WFC is crucial to mitigate physical and psychological symptoms. Implementing strategies such as workload management, stress reduction workshops, and promoting work-life balance can help enhance the well-being of female nurses. Recognizing these gender-specific patterns enables healthcare organizations to develop targeted support systems, ultimately improving the job satisfaction and retention of emergency department nurses.

## 7 Conclusion

Overall, there are differences in the effects of WFC and FWC on health outcomes among emergency department nurses of different genders. Our study confirms that WFC has a greater impact on the physical and mental well - being of female nurses, while FWC affects male nurses more significantly, suggesting implementing gender specific interventions to reduce the risk of physical and mental health issues among emergency and intensive care nurses, and addressing their symptoms. In addition, we believe that optimizing the workflow of all emergency department nurses, controlling work hours and intensity reasonably, and strengthening sleep health interventions are of great significance for improving health outcomes and stabilizing the emergency department workforce.

## Data Availability

The raw data supporting the conclusions of this article will be made available by the authors, without undue reservation.
